# Homology Modeling-Based in Silico Affinity Maturation Improves the Affinity of a Nanobody

**DOI:** 10.3390/ijms20174187

**Published:** 2019-08-27

**Authors:** Xin Cheng, Jiewen Wang, Guangbo Kang, Min Hu, Bo Yuan, Yingtian Zhang, He Huang

**Affiliations:** 1 Department of Biochemical Engineering, School of Chemical Engineering & Technology, Tianjin University, Tianjin 300350, China; 2 Key Laboratory of Systems Bioengineering (Ministry of Education), Tianjin University, Tianjin 300072, China

**Keywords:** homology modeling, in vitro affinity maturation, nanobody, rational design

## Abstract

Affinity maturation and rational design have a raised importance in the application of nanobody (VHH), and its unique structure guaranteed these processes quickly done in vitro. An anti-CD47 nanobody, Nb02, was screened via a synthetic phage display library with 278 nM of *K_D_* value. In this study, a new strategy based on homology modeling and Rational Mutation Hotspots Design Protocol (RMHDP) was presented for building a fast and efficient platform for nanobody affinity maturation. A three-dimensional analytical structural model of Nb02 was constructed and then docked with the antigen, the CD47 extracellular domain (CD47^ext^). Mutants with high binding affinity are predicted by the scoring of nanobody-antigen complexes based on molecular dynamics trajectories and simulation. Ultimately, an improved mutant with an 87.4-fold affinity (3.2 nM) and 7.36 °C higher thermal stability was obtained. These findings might contribute to computational affinity maturation of nanobodies via homology modeling using the recent advancements in computational power. The add-in of aromatic residues which formed aromatic-aromatic interaction plays a pivotal role in affinity and thermostability improvement. In a word, the methods used in this study might provide a reference for rapid and efficient in vitro affinity maturation of nanobodies.

## 1. Introduction

CD47 molecule, a ubiquitous cell-surface receptor that promotes immune evasion by interacting with signal-regulatory protein alpha (SIRPα) [1], is a member of immunoglobulin (Ig) superfamily and is considered as a promising cancer biomarker [2]. Targeted blocking CD47-SIRPα interaction for engaging macrophages to attack cancer cells represents a potentially promising immunotherapeutic strategy. Several antibody agents have been applied in clinical trials and display a positive effect [3,4]. However, there is only one approved nanobody medicine [5] and none for targeting CD47^ext^. We had screened a panel of single anti-CD47 nanobodies from a semi-synthetic library via Phage display technology (submitted work), and the optimal one (Nb02) was chosen as the parental VHH in this study.

Apart from monoclonal antibodies (mAbs), small antibody fragments of different formats have become increasingly popular binders in research and diagnostic applications due to their exceptionally high stability and selectivity [6,7,8]. Some strategies about affinity optimization were undertaken previously to improve the binding affinity and stability of biotherapeutics [9,10,11,12]. Nevertheless, given the time-consuming process of conventional approach and improved computational capabilities, structure-based computational methods involved the in silico selection of promising candidates with a high likelihood of improving binding affinity has aroused considerable interest [13,14,15,16,17]. Computational methods have been applied to improve the binding affinity, selectivity and stability of several biologics [10,18,19,20].

Nanobodies^®^ (VHHs), due to their beneficial biochemical and economic properties (small size, affinity, selectivity, stability, production cost), have been used as research tools and applied in biotechnology and medicine in recent years [6,21]. For their small mass (~125 residues and ~15 kDa) [22], the VHHs could identify some hidden antigen epitopes and infiltrate tissues more readily than mAbs [21,23,24]. On the other hand, the small size and unique structure allow it possible to utilizing computational protocols for optimizing their biophysical features, such as the binding affinity. Several applied works on computer-aided structure design [10,25,26,27] and display-based selection [28,29] have been published. Additionally, prior researches show that it takes a shorter time for in silico optimization relative to mAbs with the current computational resources [15].

For an in silico computational approach, it is significant to build a set of focused mutations and screen affinity-improved mutants accurately [9]. The ADAPT platform (Assisted Design of Antibody and Protein Therapeutics) utilizes three force-filed-based scoring functions to predict the affinity of nanobody-antigen complex. Vivcharuk et al. reported that the ADAPT platform had been proven to be practical and convenient to get affinity maturated. It was utilized for several Fab fragments affinity maturation and led to 30~100-fold affinity improvements with 50 nM~50 pM affinity constant values (*K*_D_) [30]. However, research data in ADAPT-based VHH affinity maturation are still scarce [26], and much more efforts should be made. While ADAPT-based affinity maturation owns strengths in improved affinity scoring and selection of high likelihood of improving binding, it still shows few deficiencies. The main point is the requirement of three-dimensional structural data of ADAPT-involved components. As a priori knowledge of these crystallographic structures is often not fully available, homologous modeling can be used to get the initial structure data for computational screening [31,32,33].

In the current study, we designed a fast and efficient approach to conduct Nb02′s affinity maturation. This strategy includes three significant steps: (1) homology modeling of parental VHH and initial mutant site analysis; (2) virtually screening of affinity-improved mutants using ADAPT platform; (3) binding affinity validation and molecular mechanisms analysis. In this study, we aimed to improve the affinity of the anti-CD47 VHH using homology modeling-based computational screening with experimental validation. The high-quality homology modeling structure was firstly used in ADAPT platform to replace the need for crystallographic three-dimensional (3D) structure. RMHDP (Rational Mutation Hotspots Design Protocol) determined the binding sites and used in virtual affinity maturation. ADAPT selected seven mutants, and the affinity and thermostability were tested. The best mutant (M7) showed significantly improved binding affinity with thermostability enhancement.

## 2. Results

### 2.1. Modeling and Preparation of Structure

**Figure 1:**
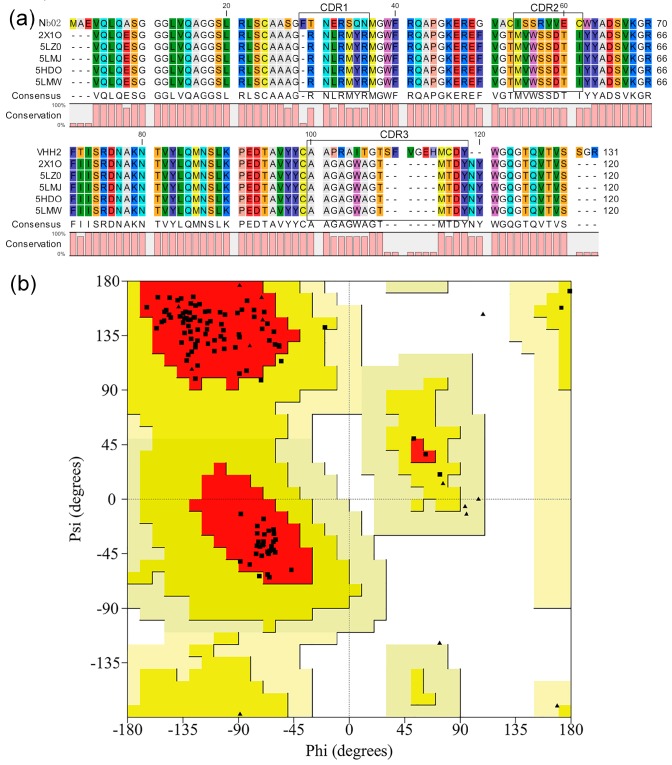
Three-dimensional (3D) structure building and assessment of Nb02. (**a**) Structural alignment of templates used in this study. (**b**) Main residues Ramachandran plot of Nb02. The squares represent the main residues, and the triangles represent G and P.

The structure of Nb02 was modeled by homology modeling using MODELLER v9.19 (http://www.salilab.org/modeller/). Five structures with the highest resolution and the lowest B-factor, namely 5LMW, 5LZ0, 5HDO, 2X1O and 5LMJ, were identified via BLAST. The sequence alignment (Figure 1) shows 26 points mutations for all the frameworks. While the side chains have different orientations, there is no difference observed in their backbone overlap. These were used as templates for modeling the Nb02. Three scoring functions (molpdf, DOPE and GA341) were used to select the optimum model. These functions are usually used to evaluate modeled structures. The molpdf and DOPE functions assess the structure’s energy and the GA341 function value determines the model’s natural state. The molpdf function is part of MODELLER software and the last two were added manually to the script. The GA341 scores are 1, which shows all models are in the natural state. The evaluation results of molpdf and DOPE are shown in Figure S1. In the model selection, we first chose five with the lowest molpdf values, and among them, the model with lowest DOPE value was chosen as the optimal one, which was model number 19. The SAVES evaluation is 100% pass, and Ramachandran plots were further used to assess and evaluate Nb02 (Figure 1b and Figure S2). Figure 1b shows the Ramachandran plot of the main residues except for G and P. Main residues located in most favored regions and additional allowed regions by 92.8% and 7.2%, respectively. In Figure S2, all 15 G residue and 3 P residues located in allowed regions. These indicate that the structure of Nb02 is rational and high-quality.

**Figure 2:**
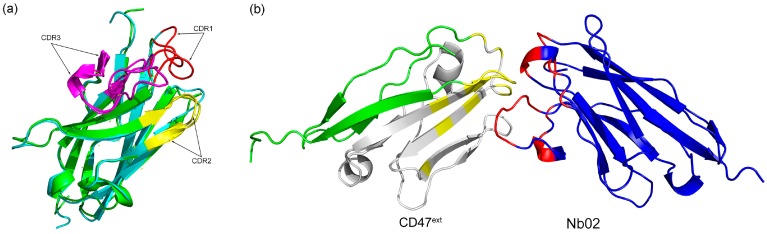
MD simulation of Nb02 and docking complex. (**a**) Nb02 end simulation conformation (green) alignment with parental VHH (cyan), CDRs are in red, yellow and purple, respectively. (**b**) Side view of Nb02-CD47^ext^ complex. The residues associated with the B6H12.2 binding sites (yellow) on the CD47^ext^ (green) and those of the CDRs (red) on the VHH (blue) is highlighted. The gray region presents the potential binding domain.

On the Nb02 structure, we ran 50 ns MD (Molecular Dynamics) simulation to eliminate unreasonable clashes and minimize energy, and analyze the whole production run and choose the lowest internal potential energy for subsequent analysis [34]. Compared to the modeled structure, significant conformational rearrangements were observed in their CDRs (Complementarity determining regions), especially with the CDR3 (as in Figure 2a). Figure S3 shows that the modeled VHH is stable over time. Along the simulated time, the backbone root means square deviation (RMSD, Figure S3a) remains smaller than 0.2 nm. Moreover, for the root mean square fluctuation (RMSF, Figure S3b), as expected, it was greater than 0.2 nm only in the predicted CDR loops, which was free to explore different conformations. As shown in Figure 2, there are also some rearrangements in backbone regions; however, the RMSD and RMSF value shows it has a much smaller impact. Moreover, nanobodies usually bind to its antigen via CDRs, especially CDR3; thus, it may be neglected in the analysis.

As experiments show that there is a competition between the VHH and the anti-CD47 mAb B6H12.2, we take the corresponding CD47^ext^ interaction sites as the CD47^ext^ active residues in this research, that is to say, the residues 29, 31, 34, 35, 37, 39, 46, 97 and 99–104 [35]. The active residues of VHH were determined as the CDRs. We then dock the Nb02 final configuration obtained after MD simulation to the selected CD47^ext^ binding sites. First, we performed a short-time pre-docking to check parameters and determine conformation rationality. After analysis of pre-docking results, we changed the docking time parameter and started production docking process. The cluster analysis and estimate results using docking score and Van der Waals energy are shown in Figure S4. We first selected three clusters with the lowest docking score and Van der Waals energy: number 1, 2 and 6. Then, the cluster with the lowest RMSD value, number 1, was chosen for subsequent analysis, as shown in Figure 2b. Comparison between different complex cluster structures indicates different binding modes of Nb02-CD47^ext^ (as shown in Figure S4d).

### 2.2. Identifying Key Residue Positions for Affinity Maturation

For affinity maturation, the identification of critical residues of the interaction between Nb02 and CD47^ext^ was crucial [10,36,37]. In the ADAPT computational approach, the first vital step is an exhaustive single-point virtual mutagenesis along the entire CDR sequence [30]. This process involved in whole CDR residues and increased the later calculation difficulty, so it is necessary to narrow down the initial interaction hotspots by a credible method, which is described in the Materials and Methods section.

As described in the Materials and Methods section, approximately 50~60% hotspots of CDRs have constant DNA sequence, which usually also encodes the antibody’s antigen-binding sites [38,39]. Moreover, the VHH has a more extended CDR3 than other antibody fragments; thus, mutagenesis aims at AGY/RGYW (R = A/G, Y = C/T and W = A/T) mutational hotspot in the CDR3 are more likely to increase the affinity. As shown in Table 1, eventually, there are six binding sites identified. Additionally, to avoid missing some residues and improving accuracy and reliability, the interface residues of Nb02 within 5 Å of CD47^ext^ were counted using PyMol, the results are also shown in Table 1.

In a word, the active residues of the Nb02 are identified: residues S55, V58, G107, T108, S109, F110, and are used as initial mutation residues for the subsequent ADAPT analysis.

### 2.3. ADAPT Cycle–multiple Mutants

In the present study, mutants were narrowed down using in silico screening and computational modeling, thus reducing in vitro analytical effort. There is a disulfide bond between C24 and C99 of Nb02, which is a hallmark of nanobody and helps to stabilize the extended CDR3 loop configuration. Therefore, each residue is mutated in turn to 17 other possible natural residues (C and P were excluded) and totaling 102 single-point mutants. In Figure S5, we show the proximity of the selected sites to the CD47^ext^.

After ADAPT round, 102 single-point mutations covered six positions that might alter antigen-binding affinity were computationally evaluated (as shown in Table S1). As described above, the top 50 single-point mutants with improved antigen-binding affinity were predicted, while utilizing a three force-filed-based scoring platform. The results are shown in Table 2.

**Figure 3:**
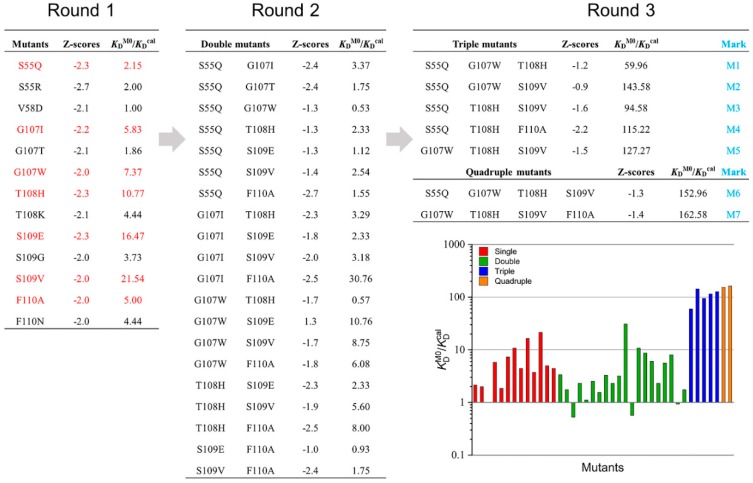
Ratios of mutant *K*_D_ relative to the parent VHH (*K*_D_^M0^/*K*_D_^cal^) during three rounds of mutations. Mutants after Round 1 that were selected forward to Round 2 are highlighted in red.

The sub-selection process is based on residue types, site diversity, and visual examination. This process narrowed down the selection to 13 mutants from the 50 top-scoring mutants for further evaluation. The CD47^ext^-binding affinities of these 13 single-point mutants were then calculated and compared with the parental VHH. The *K*_D_^cal^ and improvements in binding affinities relative to the parental VHH are presented in Figure 3. Almost all the mutants demonstrate improved binding affinities and mutations have a positive effect, excepted V58D showing no improvement. The mutant S109V is showing the highest 21.54-fold increase, with another two mutants (T108H and S109E) an over 10-fold increase. The location of these three mutations relative to the CD47^ext^ epitope is shown in Figure S5b. Additionally, mutant S109G with the substitution of residues G led to small improvement than with V and E, and T108K shows a minor increase than T108H. Mutation G107T and replacement of S55 shows only marginal gains compared to parental VHH. From the above, in this first round of ADAPT screening, there was no clear trend as to which kind of residues was more useful to the binding affinity improvement. For instance, considering the best two single-point mutants, the substitution of S109E and S109V belongs to the acidic residue and aliphatic residue, respectively, whereas the replacement of S109G had only small improvement.

Six leading single-point mutations from round 1, G107I, G107W, T108H, S109E, S109V and F110A, which possessing greater than five-fold affinity improvement in *K*_D_^cal^, were selected to round 2 affinity maturation. Despite a slight affinity improvement, the S55Q was also included in round 2 since its structural proximity to CD47^ext^ (Figure S5) and the charge-neutral mutation property. A combination of these mutant sites could lead to a total of 20 double mutants. These 20 mutants considered in this round were calculated computationally about the change in binding affinity within the ADAPT protocols, and then compared predicted affinity improvements with the component single-point mutations and parental VHH (marked as M0). There were three affinity-decreased mutants among the 20 double mutants and combined with analysis of other mutants, G107W and S109E were discarded. Consider the mutation site diversity, and after manual visual inspection of the virtual mutants, the mutations improved more than five-fold were selected to the next step. The next analysis leads to five triple mutants and two quadruple mutants (some mutants with lower improvement did not show in the figure). The seven selected multiple mutants were marked M1-M7, as shown in Figure 3. Figure S6 shows the experimentally determined and calculated $K_{D}^{M0}/K_{D}^{\mathrm{MX}}$ ratios. The experimentally determined ratios different from the calculated ones in numerical value, and this might result from the uncertainty of calculation criteria. However, the order of this value is the same, and it did not affect the selection of the best mutant.

### 2.4. Binding Affinity Validation

#### 2.4.1. Purification of VHHs

**Figure 4:**
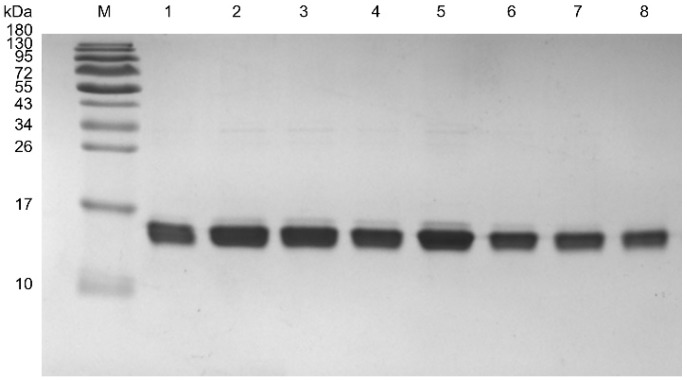
SDS-PAGE analysis for purified VHHs. M, molecular weight marker; 1–8, M0–M7. 10 μL for protein concentration 3 μg.

A HisTrap™ column was used for purifying the VHHs. The results of SDS-PAGE indicated that all of the purified VHHs showed a single band of ~15 kDa (Figure 4). Moreover, we measured the protein yield based on the purified VHHs. The yield of M0 was 1.10 mg/L, while the yield of M1–M7 were 3.20 mg/L, 2.78 mg/L, 1.92 mg/L, 3.84 mg/L, 1.46 mg/L, 1.05 mg/L and 0.60 mg/L, respectively.

#### 2.4.2. SPR

**Figure 5:**
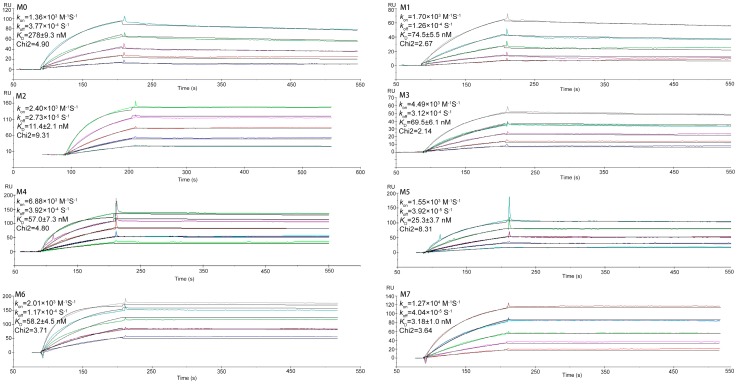
SPR sensorgram binding profiles. Interaction of the parental VHH M0 and mutants M1-M7 with immobilized CD47^ext^. The color lines represent the global fits of the raw data (black lines) to a 1:1 bimolecular model. Mean values are shown along with the VHH concentration range used in each experiment.

The *K*_D_ values were determined by SPR measurements, and the results are presented in Figure 5. By BIAcore (Biomolecular Interaction Analysis), the binding affinity of M0 was low (*K*_D_^M0^ = 278 ± 9.3 nM). To get affinity matured, we selected seven mutants by homologous modeling-based ADAPT platform. Compared with M0, the best mutation shows a 162.58-fold calculated improvement of binding affinity (*K*_D_^M0^/*K*_D_^cal^). Logically, the SPR was used to verify the *K*_D_ values and improvement folds. As shown in Figure 5, the seven mutants bound CD47^ext^ with *K*_D_ of 74.5 ± 5.5 nM, 11.4 ± 2.1 nM, 69.5 ± 6.1 nM, 57.0 ± 7.3 nM, 25.3 ± 3.7 nM, 58.2 ± 4.5 nM and 3.2 ± 1.0 nM, respectively, compared to M0. All mutants displayed a significant increase in binding affinity relative to M0, which suggests that our virtual selection protocol enables robust identification of affinity-improved mutants. With a *K*_D_ of 3.18 nM (Chi2 = 3.64), mutant M7 exhibited the greatest improvement in binding affinity of around 87.4-fold.

#### 2.4.3. qPCR

**Figure 6:**
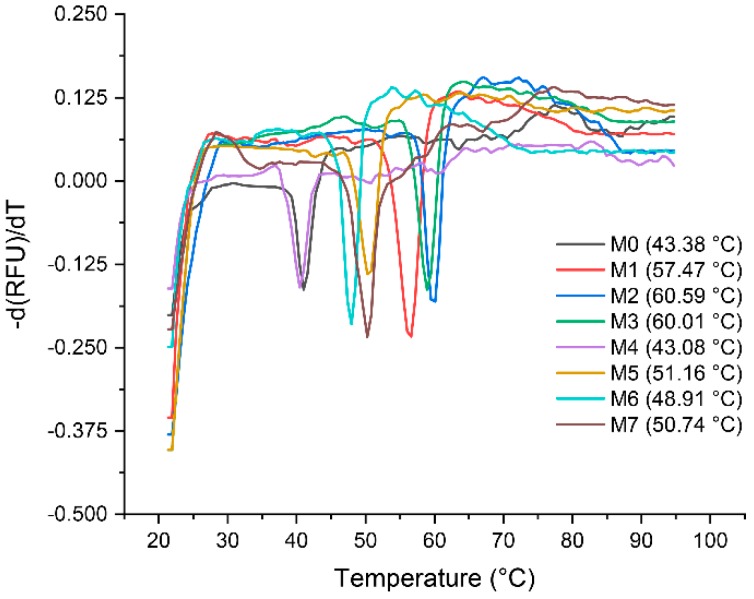
Analysis of stability of the VHHs. The values of *T*_M_ were determined by melting curves via qPCR, and each value is average from five independent experiments.

Determination of the thermal stability and melting temperatures (*T*_M_) for the seven selected mutants and parental VHH was carried out with qPCR. The results are shown in Figure 6. It shows that the melting curves of M0 and the *T*_M_ value is 43.38 °C, which is averaged from five independent experiments. The other seven mutants with *T*_M_ of 57.47 °C, 60.59 °C, 60.01 °C, 43.08 °C, 51.16 °C, 48.91 °C and 50.74 °C, respectively. With a *T*_M_ of 60.59 °C, mutant M2 shows the highest enhancement in thermal stability of 17.21 °C, while the M7 enhanced 7.36 °C. Figure S7 shows a direct comparison of affinity versus stability (*T*_M_–*K_D_*) for M0 and seven mutants.

#### 2.4.4. Indirect-ELISA by Lead Variant

**Figure 7:**
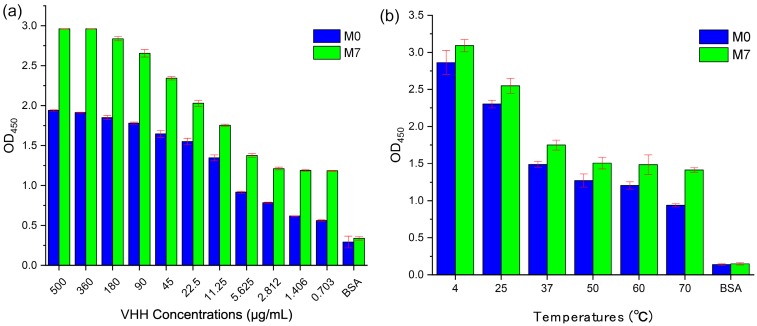
Indirect-ELISA of M0 and M7 targeting CD47^ext^. (**a**) M0/M7 were serially diluted and tested for binding to wells coated with CD47^ext^ antigen. (**b**) M0/M7 were treated 10 min in six different temperatures and added to wells coated with CD47^ext^ antigen. Bound VHHs were detected with an HRP-conjugated anti-His tag mAb, and the optical densities (OD) at 450 nm were measured by Tecan Infinite M Nano+. All samples repeated in triplicate, and values are shown as mean ± SD.

The best mutant M7 obtained at the end of the virtual screening and validated after SPR and qPCR was used to test binding activity and thermal stability with M0 through ELISA, as shown in Figure 7. Targeting CD47^ext^ in vitro binding affinity data as a function of VHH concentration are shown in Figure 7a. The best affinity-matured mutant M7 exhibited a stronger binding signal than M0 at all levels. In this assay, both VHHs show high bind ability to CD47^ext^ with the decreasing of the sample concentration. It is interesting to note that M7 can reach CD47^ext^ inhibition levels around 120.2% (at 360 μg/mL) compared to M0, whereas the value is 157.5% at 5.625 μg/mL. The BSA (Bovine Serum Albumin) was also tested as a negative control. As shown in Figure 7b, the thermostability of VHHs with different temperature treated 10 min was measured by indirect-ELISA. The data show that it still has a great binding activity to CD47^ext^ after sample treatment, and all the OD_450_ values are more than 1.0 (except OD_450_ of M0 is 0.936 at 70 °C). In particular, the OD_450_ value of M7 at 70 °C is around 45.0% compared with at 4 °C, while the value is just 32.7% for M0. These results indicate that the thermal stability of M7 is stronger than M0, and it also echoes the results of the qPCR assessment.

## 3. Discussion

A month ago, Caplacizumb was the first in class biopharmaceutical launched in both Europe and the U.S. for the treatment of aTTP (acquired Thrombotic Thrombocytopenic Purpura) [40]. Impressive progress in the application and economic outlook of nanobody has revived the researcher’s interest in developing nanobody-based biological drugs [41]. The nanobodies screened by ribosome display or phage display always possess a weaker binding affinity than mAbs, which limits its potential application in drug discovery [42,43,44]. In vivo, experimental methods such as display-based screening and enrichment based on kinds of libraries dominated the affinity maturation field [45]. With these methods, the affinity improvement could reach around 10~1000-fold [46,47,48]. However, its weakness such as being time-consuming and its randomness are also apparent and limit full application. Recent advancements in computational power, molecular dynamics (MD) trajectories and simulation have become a helpful tool to characterize the properties of protein [49]. With the increase of structural data for antibody-antigen complexes and the development and establishment of algorithms for binding affinity prediction, the computer-aided structure-based rational approach has been an attractive choice for antibody affinity maturation. Currently, the dominating limitation of ADAPT is the need for a pre-existing crystal structure of the target antibody-antigen complex, even though the ADAPT platform could bring an improvement around 10–100-fold range based on several limited applications. Ideally, it could be replaced by a high-quality model based on homologous structure relative to the parental sequence.

The primary consideration of this study is to develop an efficient, engineered, and universal protocol for the in vitro affinity maturation of nanobodies to lay the foundation for drug development.

The parental VHH of this study was selected via three rounds of panning of a synthetic nanobody library, which display a weak binding affinity targeting CD47^ext^ (*K*_D_ = 278 nM). Initial concerns emerged due to the uncertainty of created strictly homologous structure compared to the actual fabric. Here, we build its 3D structure by MODELLER based on five homologous crystal nanobody structures. Ramachandran plots (Figure 1b and Figure S2) and MD simulation results (Figure 2 and Figure S3) show that the structures of Nb02 were rational and high-quality. The Nb02 was then utilized in ADAPT-based virtual affinity maturation, with the replacement of crystal structure, and the experimental validation of mutants shows a significant improvement in binding affinity and thermostability.

One limitation of the ADAPT protocol is the inadequate conformational sampling of the binding between the mutants and the target protein, essential for an accurate estimation of the binding affinity. This affinity maturation protocol approximates that the changes in the binding conformation after a single mutation are negligible. To observe these conformational changes, an MD simulation of each new complex was performed. The structure aligns results of M1~M7 show that their binding does not suffer great rearrangements.

**Figure 8:**
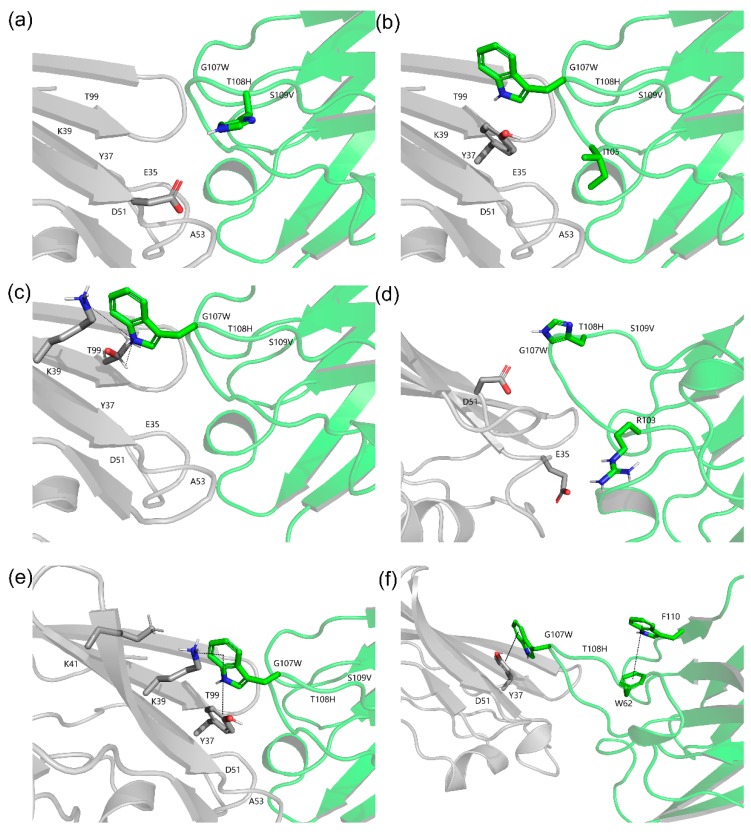
Structure models of optimized CD47^ext^-VHH interactions. The VHH and CD47^ext^ chains are colored green and grey, respectively. The corresponding C atoms are colored blue and H atoms colored red. (**a**) Structure model of the T108H. A salt bridge is formed between T108H and 51D of CD47^ext^. (**b**) Structure model of the G107W. Hydrophobic interactions are created between G107W and 37Y of CD47^ext^. (**c**) Hydrogen bonds formed by G107W. H-bonds are shown in black and dashed lines. (**d**) Ionic interactions built by T108H. (**e**) Cation-Pi interaction (G107W-39K/41K) and Aromatic-Aromatic interaction (G107W-37Y). (**f**) Aromatic-Aromatic interaction of F110 and G107W.

Rational structure-based affinity maturation contributes to an understanding of the structural basis for the improvement of binding affinity, which is one of its advantages. Figure 8 displays the structural basis and interactions predicted for quadruple mutant M7 (G107W, T108H, S109V, F110A) with CD47^ext^ [50]. The substitution of residue T108 by H introduce a salt bridge with D51 on CD47^ext^ (Figure 8a). Furthermore, two sets of ionic interaction formed between T108H and D51, E35 and R103 of VHH. On the G107 site, mutation to W generate a hydrophobic interaction with Y37 of antigen with a distance 2.74 Å (Cα–Cα); relatively, the distance of previous Y37 hydrophobic interaction with I105 of VHH is 5.51 Å (Figure 8b). Additionally, the mutation of G107W constitutes hydrogen bonds: the main chain-side chain H-bound between K39 and G107W, the side chain-side chain H-bound between G107W and T99 (Figure 8c). The new intramolecular H-bound interactions introduced by W at position 107 are also likely contributed to the observed improvement of binding affinity. In Figure 8e, it shows there still formed a cation-Pi interaction between G107W and K39 (4.63 Å) and K41 (5.13 Å).

Another critical factor is the add-in of aromatic residue, which is beneficial to protein structure stabilization [51]. The aromatic residue W interacted with cation provided by electro-positive residue K and formed a stronger bond than a salt bridge, which could also be likely responsible for affinity improvement. Additionally, the mutation of G107 to W introduces a new aromatic-aromatic interaction with Y37 (4.65 Å) of CD47^ext^ (Figure 8e). The structure model shows that the F110 formed an aromatic-aromatic interaction with W62 neared CDR2 (Figure 8f). From above, we can see that even on the same site, there could be different mechanisms of improving binding affinity. Hence, the result figures out the importance of aromatic residue in the enhancement of affinity and stability. Inoue et al. carried out an in silico virtual analysis of a CDR3-grafted VHH and obtained a mutant that had a ~20-fold improvement over the original protein as measured by SPR [10]. Their work and some others are all substitutions by charged residues, such as R and D. In contrast, the designed mutants in this study have informative ways in affinity improvement as they mutated to several kinds of residues, such as aromatic G107W and aliphatic F110A, and did not solely rely on electrostatics (alkaline T108H).

Taken together, our finding demonstrates that high-quality homology structure can afford ADAPT-guided binding affinity improvements. These data also show that ADAPT-guided affinity maturation of VHH for improved antigen-binding ability can also translate into an enhancement of thermostability. The combination of improved affinity and thermostability is commonly tricky during the process of nanobody affinity maturation. Prior research has shown that increased affinity results from mutations of CDRs sites always accompanied by structure destabilizing [26,30]. This destabilization may attribute to the change of strain of framework results from the residue replacement and corresponding structure modification. Another likely reason might be the change of chemical property of antigen-binding sites selected for affinity maturation [37]. Encouragingly, our six selected multiple mutants displayed addition stability (except M4) and the highest affinity increased variant M7 with 7.36 °C enhancement of *T*_M_ value was identified by qPCR (*T*_M_ of 50.74 °C relatives to 43.38 °C of M0). In detail, the M4 and M7, which include F110A mutation, have a weaker stability enhancement than others, the *T*_M_ of M4 even smaller than M0. The most probable cause is that the six antigen-binding sites for affinity maturation are all located in the CDRs region, and its substitutions scarcely affect the whole stability. Another reason, as above, is the aromatic residue. The side chains of aromatic residue, which possess directional Pi-systems, always stabilize protein interiors and interface. Thus, substitution by aromatic residue could enhance the structural stability of protein [52,53], and in this study is F and W. Nevertheless, antibodies aggregate at high temperatures and thus may influence the accurate determination of their *T*_M_ value [54]. Admittedly, the stability improvement observed by thermal denaturation might occur due to delayed onset of aggregation.

This study reveals several key factors which may affect the efficiency and robustness during nanobody in silico affinity maturation. First, these results illuminated that multiple mutations are necessary to achieve a considerable improvement in binding affinity for the anti-CD47 VHH. Previous works demonstrate that multi-single point affinity-matured mutants result in a moderate increase in binding affinity of multiple mutations [10,25,26,30], while our finding is generally consistent with it. It is possible and relatively straightforward to identify and combine several affinity-enhanced single mutations; nevertheless, the additive effects of multiple mutations on nanobody affinity are complex and incompletely understood thus far. Additionally, it sometimes hurts binding affinity [55,56]. When ADAPT platform was used in Fab affinity maturation, the first step is an exhaustive single-point virtual mutagenesis along the entire CDR sequence. It is time-consuming and abnormal to generate all possible combinations of single-point mutations, which led to a large amount of affinity evaluation and multiple-rounds of screening. Moreover, nanobody has a more extended CDR1 and CDR3 than the traditional antibody. These two factors determined that it is challenging to utilize ADAPT screening platform with whole CDR. As described in Materials and Methods section, the binding sites were identified by a Rational Mutation Hotspots Design Protocol (RMHDP). Each has different predicted binding sites, and we selected the overlapped parts as a credible and rational result. This method immensely narrowed down the initial mutagenesis amounts (17 mutations per CDR site across six sites in this study). These sites were utilized in virtual affinity maturation and eventually led to positive and encouraging results, as shown above.

Technically, virtual screening filtered the best mutant among all virtual candidates in the development to affinity-improved mutant and hence moved ahead of the affinity validation study. In this study, we undertook the preparation of a nanobody structure by homologous modeling. Compared to crystallography-determined structure data, the use of homologous modeling considerably lowering the hurdle to conduct affinity maturation in vitro to some degree. However, comparable data is still scarce, and further studies are still necessary. Furthermore, additional experiments will be based on synthetic biology concepts to rationally design bispecific nanobodies to enhance the affinity and targeting the ability of nanobody-based biotherapeutics. Moreover, the effect of the drug increases the phagocytic capacity of macrophages to cancer cells will be tested, while the antitumor effect on an animal model of carcinoma in situ.

## 4. Materials and Methods

### 4.1. Homology Modeling

The sequence of Nb02 (GenBank accession number MK780744) and CD47^ext^ are shown in Figure S8. The sequence of the Nb02 used for experimental validation in this study has a C-terminal His6 tag that was not included for in silico studies. We used the MODELLER v9.19 to build the structural models of Nb02. The experimentally determined sequences were searched in the protein databank with BLAST (http://blast.ncbi.nlm.nih.gov/Blast.cgi), E-value cutoff 10.0 and sequence identity cutoff 90%. The resulting nanobody sequences were aligned with CLC Sequence Viewer 7.8.1 (CLC bio, Finlandsgade, Aarhus N, Denmark). The highest resolution and the lowest B-factor of the results, namely 5LMW [57], 5LZ0 [57], 5HDO [58], 2X1O [59] and 5LMJ [57], were chosen as templates. First, 50 models were built and the optimum one was selected based on the use of three different criteria (molpdf, DOPE and GA341). After that, it was evaluated and optimized using the webserver SAVES (http://services.mbi.ucla.edu/SAVES/), and then assessed using the Ramachandran plot. Eventually, an MD simulation was performed to eliminate unreasonable structure clash and minimize the energy.

### 4.2. MD Simulation

All MD simulations and their analysis were run as implemented in the GROMACS package (http://www.gromacs.org) [60] with the AMBER99SB-ILDN42 force field [61] and SPC water. The Nb02–CD47^ext^ complexes were first minimized without solvent, and then placed the molecules in a cubic box with a water layer of 1.2 nm and the system was neutralized by adding two Cl^−^ as counterions. Subsequently, a second minimization of the system was performed before starting the MD simulation. After minimization, the equilibrium was performed for 100 ps in the NVT ensemble, followed by 20 ns NPT production run. The temperature was controlled at 300 K by a modified Berendsen thermostat [62], with a time constant of 0.5 ps. The pressure was controlled at 1 bar by isotropic Parrinello–Rahman method [63] with coupling constants of 1.0 ps.

Moreover, a Periodic boundary condition was used for all MD simulations. The iteration time step was set to 2 fs with the Verlet integrator and LINCS (Linear Constraint Solver) constraints [64]. The MD simulations were then performed for 50 ns.

### 4.3. Molecular Docking

Through computational analysis combined with analysis of solvent-exposed hydrophobic regions, the active residues of the Nb02 are identified: residues 55, 58, 107–110. Additionally, passive residues were automatically defined. Molecular docking using AutoDock Vina (http://vina.scripps.edu/) [65] and its standard protocols were conducted to predict the Nb02-CD47^ext^ complex structure. The atomic coordinates of the CD47^ext^ were taken from the structure of the SIRPα-CD47 complex crystallized at pH 8.5 (PDB ID: 4CMM) [66]. Active residues were defined for each VHH as mentioned above, while CD47^ext^ active residues were those known to be in contact with the commercially available anti-CD47 mAb B6H12.2: residues 29, 31, 34, 35, 37, 39, 46, 97 and 99–104 [35]. While other parameters were set to the default values, the size of the grid box was 40 Å × 40 Å × 40 Å, and the Lamarckian genetic algorithm was used to search the complex pose. Among the docking results obtained, we chose the docking cluster with the lowest docking score and binding free energy for the top conformations.

### 4.4. Rational Mutation Hotspots Design Protocol

Two different criteria are used to identify and pre-select the interface residues which might affect the affinity. First, the CDR residues within a contact distance with the antigen might affect the binding affinity. Thus, we counted the surface residue residues of parental VHH within a 5 Å distance of CD47^ext^ using PyMol (http://www.pymol.org/). Second, mutations of variable regions in the affinity maturation frequently belong to some constant CDR sequences, namely “Hot Spots”, which residues generally were AGY or RGYW (R = A or G, Y = C or T,W = A or T) [38,39,67,68]. These residues are also often located at antigen-binding sites for antibodies, and mutations aim at these sites is more likely than others to improve the binding affinity. The overlapped parts are selected as a credible and rational result of rational mutation hotspots design. The mutations were done with Swiss-PDB Viewer 4.1 (http://spdbv.vital-it.ch/) [69], and all obtained models underwent an MD simulation.

### 4.5. In Silico Affinity Maturation

It has been reported that a few residues’ changes could significantly improve the binding affinity of proteins in a previous publication [10]. In the present study, ADAPT was used to optimize the binding affinity of Nb02. A 3D structure model of the antibody-antigen complex is necessary for the ADAPT calculation. In this study, the initial sequence of the Nb02 was screened from a semi-synthetic library via Phage display technology (submitted work). The protein structure of Nb02 is predicted by homology modeling using the protocol described above and were used as a starting point for virtual affinity maturation.

Some structural preprocessing had to be done before the computational screening. Initially, water molecule and counterions of the VHH modeling structure were removed. Secondly, hydrogen atoms were added and adopted to protonation states at neutral pH. Thirdly, structural refinement was carried out by energy-minimization using the AMBER force-field, with a distance-dependent dielectric (4*r*_ij_) and an infinite distance cutoff for non-bonded interactions. The non-hydrogen atoms were restrained at their initial positions with a harmonic force constant of 10 kcal/(mol·A^2^).

In the first round of affinity optimizations, six positions (S55, V58, G107, T108, S109, F110) within the CDRs of the parental sequence were mutated to 17 other possible natural residues (Cys and Pro were excluded). 102 single-point scanning mutagenesis was carried out in this step. Three protocols, SIE-SCWRL, FoldX and Rosetta, were used to building and repacking the structures and evaluating the energies of these single-point mutations. Each component energy function would give a Z-score about the binding affinity of these mutants, and a consensus Z-score was further applied for scoring these mutants to alleviate the influence of the median and median absolute deviation. For the ratio of improved binding affinity, $K_{D}^{M0}/K_{D}^{\mathrm{cal}}$, we employed HADDOCK webserver and Schrodinger to calculate the *K*_D_ values. Further technical and implementation details of this consensus approach and its component methods can be found in Sulea et al. [26]. Consider the mutation site diversity, and after manual visual inspection of the virtual mutants, approximately 15 mutants from the 50 top-scoring mutants are selected for further optimizations. In the second round of optimization, combine the favorable single-point mutations with Z-score using the same computational protocol above, a series of double- and triple-point VHH mutants were generated, and so do the quadruple-point mutants’ generation.

### 4.6. Protein Expression and Purification

The DNA sequences of VHH mutants were synthesized commercially by GENEWIZ (Beijing, China), subcloned into the pET-32a (+) expression plasmid, with N-terminal RBS/TATA box and C-terminal His6 tag, and were subsequently expressed in *Trans*B (DE3) *E. coli*. For expression, 2 mL of the overnight culture was inoculated into LB media (200 mL) supplemented with 100 µg/mL ampicillin in 500 mL flasks with air top seals and grown until an OD_600_ ~0.6–0.8. Cultures were then induced with 200 µL IPTG (500 mM) and grown for 16 h at 18 °C with shaking at 180 rpm. After that, the interest proteins were extracted by sonication and centrifugation, and the supernatants get filtered through 0.45µM filters. The purification of VHHs was carried out by employing Ni^2+^-affinity chromatography (IMAC) using HisTrap™ HP (GE Healthcare, Tianjin, China). The binding buffer containing PBS pH 7.4, 500 mM NaCl, 80 mM imidazole, and the elution buffer containing PBS pH 7.4, 500 mM NaCl, 500 mM imidazole. The recombinant monomeric protein was visualized by SDS-PAGE.

### 4.7. SPR

The IMAC-purified VHHs were subjected to desalting and soluble in PBS (pH 7.4) before the SPR binding experiments. Binding interactions were conducted with an SPR-based Biacore 3000 instrument (GE Healthcare) at 25 °C. Protein concentrations were quantified by 280 nm absorbance with a Nanodrop2000 spectrometer (Thermo Scientific, Shanghai, China). Typically, approximately 350 resonance units (RUs) of enzymatically biotinylated CD47^ext^ (ab174029, Abcam, Shanghai, China) were directionally immobilized on a CM5 sensor chip (GE Healthcare). An unrelated biotinylated protein was immobilized with an RU value matching that of the reference surface to control for nonspecific binding. Measurements were made with serial VHH concentrations in Biacore running buffer (10 mM HEPES, 150 mM NaCl, 3 mM EDTA, 0.05%(*v*/*v*) P20, pH 7.4; GE Healthcare) at a flow rate of 40 μL/min. Running buffer without VHH was then passed over the chip to allow spontaneous dissociation at the same flow rate. The CD47 surface was regenerated by three 60 s injections of 10 mM Glycine-HCl buffer (pH 2.1) after each sample injection. All data were analyzed with the Biacore 3000 evaluation software (GE Healthcare) with a 1:1 Langmuir binding model. All VHHs were run in duplicates.

### 4.8. Thermal Stability Measurements

To investigate the thermal stability changes of VHHs, real-time fluorescence quantitative PCR (qPCR) was used to measuring the melting curves of the parental and mutant VHHs, and the melting temperatures (*T*_m_) were acquired by taking a negative first derivative transformation [70]. qPCR was carried out in a Roche LightCycler^®^ 480 II real-time PCR instrument (country Roche, Beijing, China). A total volume of 20 μL in each tube of 96-well plates was used. 1.0 μL HEPES (1 M) and 0.04 μL TCEP (0.25 M) was added first. Samples were diluted in PBS at a final concentration, after mixing, of 2.0 μM. 17.46 μL 1× PBS was then added. SYPRO^®^ Orange (Roche, Beijing, China) was diluted 25-fold from the 5000× concentrated stock to the working dye solution in PBS, and 0.5 μL was added to the mixture just before the experiment. Thermal denaturation temperature was ranged from 25 °C to 95 °C at a rate of 0.01 °C/s. Fluorescence intensity, with excitation at 465 nm and emission at 580 nm, was collected at 1 °C intervals and analyzed with the correlative software Exor4 (Roche Applied Science) and LightCycler Thermal Shift Analysis. Each sample was measured in quintuplicate and the average arithmetic value was calculated.

### 4.9. Indirect ELISA

The specificity of M0 and selected indirect ELISA assessed the best mutant with indirectly coated CD47^ext^/VHH complex in 96-well plates, where the bound VHH was detected using an HRP-conjugated anti-His tag mAb. The CD47^ext^ and BSA were diluted to 30 μg/mL and coated overnight at 4 °C. After PBST wash, add 200 μL blocking liquid and incubated with 2.5 h at 37 °C. For binding affinity test, add 100 μL VHH and incubated with 2.5 h at 37 °C. As for thermal sensitivity experiment, add 100 μL VHH with diluted concentration 30 μg/mL and incubated with 2 h at 37 °C. After anti-His tag mAb added, the plates were then stained with TMB (Solarbio^®^, Beijing, China), and the OD_450_ values were immediately measured in Infinite M Nano^+^ (Tecan, Tianjin, China). For the thermal stability assessment, the VHHs were treated with a different temperature for 10 min rather than diluted to serial concentrations. BSA was used as negative control. Origin 2018 was used for the representation of all ELISA data.

## 5. Conclusions

In summary, the combination of in silico screening of VHH mutants and experimental measurements may be used for the estimation of the relative binding affinity maturation in similar protocols for the design and optimization of VHHs as binders of protein targets. The fast and efficient approach designed in this study, which uses homologous modeling structure in ADAPT platform to screen multiple mutations, is useful for increasing the binding affinity of a phage display VHH while enhancing stability. These results will need to be assessed for other VHHs to evaluate their commonality. With the development of computational power and algorithms’ optimization, there is still room for further improvement in the accuracy and usability of virtual mutation screening. The structure features of nanobody guaranteed in silico affinity maturation done in a fast and convenient way. Moreover, the RMHDP utilized in optimizing binding sites identification was efficient and rational, which turns out to be valid to affinity improvement. It reduced unnatural residues and additional calculation, to overcome in silico affinity maturation period to 3~4 weeks from more than two months. These two points form the foundation of our computational approach. The platform described in this study for in silico nanobody affinity maturation based on homology modeling, the RMHDP and ADAPT will be further optimized. The beneficial properties of nanobody provide rapid development of nanobody-based high-affinity binders and offer new ideas for the treatment of “orphan” diseases and explosive infectious diseases.

## Figures and Tables

**Table 1:** Binding sites analyses of anti-CD47 VHH using two criteria ^1^.

Interface Residues Analysis		AGY/RGYW	
32	GLU (E)	34	SER (S)
35	GLN (Q)	55	SER (S)
36	ASN (N)	56	SER (S)
54	ILE (I)	58	VAL (V)
55	SER (S)	59	VAL (V)
57	ARG (R)	100	ALA (A)
58	VAL (V)	101	ALA (A)
60	GLU (E)	107	GLY (G)
61	CYS (C)	108	THR (T)
62	TRP (W)	109	SER (S)
105–113	-	110	PHE (F)

^1^ The overlapped residues are selected as a credible and rational result of rational mutation hotspots design. For both two panel, the left column represents the residue number and right column for corresponding residue name.

**Table 2:** Top 50 consensus Z-scores (<0) for single mutants ^1^.

Res	A	R	N	D	Q	E	G	H	I	L	K	M	F	S	T	W	Y	V
S55		−2.7	−1.7	−2.2	−2.3					−2.1			−1.8				−1.7	
V58			−1.7	−2.1						−1.9	−1.7	−1.7		−1.7	−1.7			
G107		−2.1			−1.9	−1.8		−1.7	−2.2	−1.8	−1.8	−1.8	−1.7	−1.9	−2.1	−2.0	−1.9	−1.7
T108								−2.3			−2.1		−1.7			−1.9	−1.8	−1.7
S109			−1.6	−2.0		−2.3	−2.0				−1.6		−1.9			−1.7		−2.0
F110	−2.0	−2.2	−2.0		−1.7	−1.6				−1.7				−1.9				−1.6

^1^ 13 single-point mutations selected for Round 2 are highlighted in red. Nb02 Z-score = −0.20.

## Supplementary Materials

Supplementary Materials can be found at https://www.mdpi.com/1422-0067/20/17/4187/s1.

## Abbreviations

*K*_D_	Dissociation constant
*T*_M_	Melting Temperature
RMHDP	Rational Mutation Hotspots Design Protocol
VHHs	Single domain antibodies
CD47ext	CD47 extracellular domain
mAbs	Monoclonal antibodies
ScFvs	Single-chain variable-fragments
ADAPT	Assisted Design of Antibody and Protein Therapeutics
MD	Molecule Dynamics
CDRs	Complementarity determining regions
OD450	Optical Densities at 450 nm
aTTP	acquired Thrombotic Thrombocytopenic Purpura
DOPE	Discrete Optimized Protein Energy
MD	Molecular Dynamics
BSA	Bovine Serum Albumin
HRP	Horseradish Peroxidase
NVT	Number of particles, Volume, Temperature
NPT	Number of particles, Pressure, Temperature
SPR	Surface Plasmon Resonance
qPCR	quantitative Polymerase Chain Reaction

## References

[1] Barclay A.N. (2009). Signal regulatory protein alpha (SIRPalpha)/CD47 interaction and function. Curr. Opin. Immunol..

[2] Zhang L., Huang H. (2016). Targeting the Cancer Biomarker CD47: A Review on the Diverse Mechanisms of the CD47 Pathway in Cancer Treatment. Anticancer Agents Med. Chem..

[3] Ngo M., Han A., Lakatos A., Sahoo D., Hachey S.J., Weiskopf K., Beck A.H., Weissman I.L., Boiko A.D. (2016). Antibody Therapy Targeting CD47 and CD271 Effectively Suppresses Melanoma Metastasis in Patient-Derived Xenografts. Cell Rep..

[4] Liu J., Wang L., Zhao F., Tseng S., Narayanan C., Shura L., Willingham S., Howard M., Prohaska S., Volkmer J. (2015). Pre-Clinical Development of a Humanized Anti-CD47 Antibody with Anti-Cancer Therapeutic Potential. PLoS ONE.

[5] Scully M., Cataland S.R., Peyvandi F., Coppo P., Knobl P., Kremer Hovinga J.A., Metjian A., de la Rubia J., Pavenski K., Callewaert F. (2019). Caplacizumab Treatment for Acquired Thrombotic Thrombocytopenic Purpura. N. Engl. J. Med..

[6] Allegra A., Innao V., Gerace D., Vaddinelli D., Allegra A.G., Musolino C. (2018). Nanobodies and Cancer: Current Status and New Perspectives. Cancer Investig..

[7] Kandalaft H., Hussack G., Aubry A., van Faassen H., Guan Y., Arbabi-Ghahroudi M., MacKenzie R., Logan S.M., Tanha J. (2015). Targeting surface-layer proteins with single-domain antibodies: A potential therapeutic approach against Clostridium difficile-associated disease. Appl. Microbiol. Biotechnol..

[8] McCafferty J., Schofield D. (2015). Identification of optimal protein binders through the use of large genetically encoded display libraries. Curr. Opin. Chem. Biol..

[9] Tiller K.E., Tessier P.M. (2015). Advances in Antibody Design. Annu. Rev. Biomed. Eng..

[10] Inoue H., Suganami A., Ishida I., Tamura Y., Maeda Y. (2013). Affinity maturation of a CDR3-grafted VHH using in silico analysis and surface plasmon resonance. J. Biochem..

[11] Park S.G., Lee J.S., Je E.Y., Kim I.J., Chung J.H., Choi I.H. (2000). Affinity maturation of natural antibody using a chain shuffling technique and the expression of recombinant antibodies in *Escherichia coli*. Biochem. Biophys. Res. Commun..

[12] Cumbers S.J., Williams G.T., Davies S.L., Grenfell R.L., Takeda S., Batista F.D., Sale J.E., Neuberger M.S. (2002). Generation and iterative affinity maturation of antibodies in vitro using hypermutating B-cell lines. Nat. Biotechnol..

[13] Sudha G., Nussinov R., Srinivasan N. (2014). An overview of recent advances in structural bioinformatics of protein-protein interactions and a guide to their principles. Prog. Biophys. Mol. Biol..

[14] Yugandhar K., Gromiha M.M., Zhou Y., Kloczkowski A. (2017). Computational Approaches for Predicting Binding Partners, Interface Residues, and Binding Affinity of Protein–Protein Complexes. Prediction of Protein Secondary Structure.

[15] Soler M.A., Fortuna S., de Marco A., Laio A. (2018). Binding affinity prediction of nanobody-protein complexes by scoring of molecular dynamics trajectories. Phys. Chem. Chem. Phys..

[16] Sirin S., Apgar J.R., Bennett E.M., Keating A.E. (2016). AB-Bind: Antibody binding mutational database for computational affinity predictions. Protein Sci..

[17] Pires D.E., Ascher D.B. (2016). mCSM-AB: A web server for predicting antibody-antigen affinity changes upon mutation with graph-based signatures. Nucleic Acids Res..

[18] Kiyoshi M., Caaveiro J.M., Miura E., Nagatoishi S., Nakakido M., Soga S., Shirai H., Kawabata S., Tsumoto K. (2014). Affinity improvement of a therapeutic antibody by structure-based computational design: Generation of electrostatic interactions in the transition state stabilizes the antibody-antigen complex. PLoS ONE.

[19] Lin S.G., Ba Z., Du Z., Zhang Y., Hu J., Alt F.W. (2016). Highly sensitive and unbiased approach for elucidating antibody repertoires. Proc. Natl. Acad. Sci. USA.

[20] Fennell B.J., McDonnell B., Tam A.S., Chang L., Steven J., Broadbent I.D., Gao H., Kieras E., Alley J., Luxenberg D. (2013). CDR-restricted engineering of native human scFvs creates highly stable and soluble bifunctional antibodies for subcutaneous delivery. MABS.

[21] Muyldermans S. (2013). Nanobodies: Natural single-domain antibodies. Annu. Rev. Biochem..

[22] Hamers-Casterman C., Atarhouch T., Muyldermans S., Robinson G., Hamers C., Songa E.B., Bendahman N., Hamers R. (1993). Naturally occurring antibodies devoid of light chains. Nature.

[23] Zavrtanik U., Lukan J., Loris R., Lah J., Hadži S. (2018). Structural Basis of Epitope Recognition by Heavy-Chain Camelid Antibodies. J. Mol. Biol..

[24] Mitchell L.S., Colwell L.J. (2018). Analysis of nanobody paratopes reveals greater diversity than classical antibodies. Protein Eng. Des. Sel..

[25] Yau K.Y., Dubuc G., Li S., Hirama T., Mackenzie C.R., Jermutus L., Hall J.C., Tanha J. (2005). Affinity maturation of a V(H)H by mutational hotspot randomization. J. Immunol. Methods.

[26] Sulea T., Hussack G., Ryan S., Tanha J., Purisima E.O. (2018). Application of Assisted Design of Antibody and Protein Therapeutics (ADAPT) improves efficacy of a Clostridium difficile toxin A single-domain antibody. Sci. Rep..

[27] Hussack G., Riazi A., Ryan S., van Faassen H., MacKenzie R., Tanha J., Arbabi-Ghahroudi M. (2014). Protease-resistant single-domain antibodies inhibit *Campylobacter jejuni* motility. Protein Eng. Des. Sel..

[28] McMahon C., Baier A.S., Pascolutti R., Wegrecki M., Zheng S., Ong J.X., Erlandson S.C., Hilger D., Rasmussen S.G.F., Ring A.M. (2018). Yeast surface display platform for rapid discovery of conformationally selective nanobodies. Nat. Struct. Mol. Biol..

[29] Uchański T., Zögg T., Yin J., Yuan D., Wohlkönig A., Fischer B., Rosenbaum D.M., Kobilka B.K., Pardon E., Steyaert J. (2019). An improved yeast surface display platform for the screening of nanobody immune libraries. Sci. Rep..

[30] Vivcharuk V., Baardsnes J., Deprez C., Sulea T., Jaramillo M., Corbeil C.R., Mullick A., Magoon J., Marcil A., Durocher Y. (2017). Assisted Design of Antibody and Protein Therapeutics (ADAPT). PLoS ONE.

[31] Soler M.A., de Marco A., Fortuna S. (2016). Molecular dynamics simulations and docking enable to explore the biophysical factors controlling the yields of engineered nanobodies. Sci. Rep..

[32] Russo A., Scognamiglio P.L., Hong Enriquez R.P., Santambrogio C., Grandori R., Marasco D., Giordano A., Scoles G., Fortuna S. (2015). In Silico Generation of Peptides by Replica Exchange Monte Carlo: Docking-Based Optimization of Maltose-Binding-Protein Ligands. PLoS ONE.

[33] Soler M.A., Rodriguez A., Russo A., Adedeji A.F., Dongmo Foumthuim C.J., Cantarutti C., Ambrosetti E., Casalis L., Corazza A., Scoles G. (2017). Computational design of cyclic peptides for the customized oriented immobilization of globular proteins. Phys. Chem. Chem. Phys..

[34] Perricone U., Gulotta M.R., Lombino J., Parrino B., Cascioferro S., Diana P., Cirrincione G., Padova A. (2018). An overview of recent molecular dynamics applications as medicinal chemistry tools for the undruggable site challenge. Medchemcomm..

[35] Pietsch E.C., Dong J., Cardoso R., Zhang X., Chin D., Hawkins R., Dinh T., Zhou M., Strake B., Feng P.H. (2017). Anti-leukemic activity and tolerability of anti-human CD47 monoclonal antibodies. Blood Cancer J..

[36] Gonzalez-Munoz A., Bokma E., O’Shea D., Minton K., Strain M., Vousden K., Rossant C., Jermutus L., Minter R. (2012). Tailored amino acid diversity for the evolution of antibody affinity. MABS.

[37] Akiba H., Tsumoto K. (2015). Thermodynamics of antibody-antigen interaction revealed by mutation analysis of antibody variable regions. J. Biochem..

[38] Jolly C.J., Wagner S.D., Rada C., Klix N., Milstein C., Neuberger M.S. (1996). The targeting of somatic hypermutation. Semin. Immunol..

[39] Goyenechea B., Milstein C. (1996). Modifying the sequence of an immunoglobulin V-gene alters the resulting pattern of hypermutation. Proc. Natl. Acad. Sci. USA.

[40] Peyvandi F., Scully M., Kremer Hovinga J.A., Knobl P., Cataland S., De Beuf K., Callewaert F., De Winter H., Zeldin R.K. (2017). Caplacizumab reduces the frequency of major thromboembolic events, exacerbations and death in patients with acquired thrombotic thrombocytopenic purpura. J. Thromb. Haemost..

[41] Siontorou C.G. (2013). Nanobodies as novel agents for disease diagnosis and therapy. Int. J. Nanomed..

[42] Wesolowski J., Alzogaray V., Reyelt J., Unger M., Juarez K., Urrutia M., Cauerhff A., Danquah W., Rissiek B., Scheuplein F. (2009). Single domain antibodies: Promising experimental and therapeutic tools in infection and immunity. Med. Microbiol. Immunol..

[43] Stewart C.S., MacKenzie C.R., Hall J.C. (2007). Isolation, characterization and pentamerization of alpha-cobrotoxin specific single-domain antibodies from a naive phage display library: Preliminary findings for antivenom development. Toxicon.

[44] Wagner H.J., Wehrle S., Weiss E., Cavallari M., Weber W. (2018). A Two-Step Approach for the Design and Generation of Nanobodies. Int. J. Mol. Sci..

[45] Qiao C.X., Lv M., Li X.Y., Geng J., Li Y., Zhang J.Y., Lin Z., Feng J.N., Shen B.F. (2013). Affinity maturation of antiHER2 monoclonal antibody MIL5 using an epitope-specific synthetic phage library by computational design. J. Biomol. Struct. Dyn..

[46] Yu X., Qu L., Bigner D.D., Chandramohan V. (2017). Selection of novel affinity-matured human chondroitin sulfate proteoglycan 4 antibody fragments by yeast display. Protein Eng. Des. Sel..

[47] Liu W., Song H., Chen Q., Yu J., Xian M., Nian R., Feng D. (2018). Recent advances in the selection and identification of antigen-specific nanobodies. Mol. Immunol..

[48] Wang Y., Keck Z.Y., Saha A., Xia J., Conrad F., Lou J., Eckart M., Marks J.D., Foung S.K. (2011). Affinity maturation to improve human monoclonal antibody neutralization potency and breadth against hepatitis C virus. J. Biol. Chem..

[49] Yamashita T. (2018). Toward rational antibody design: Recent advancements in molecular dynamics simulations. Int. Immunol..

[50] Tina K.G., Bhadra R., Srinivasan N. (2007). PIC: Protein Interactions Calculator. Nucleic Acids Res..

[51] Burley S., Petsko G. (1985). Aromatic-aromatic interaction: A mechanism of protein structure stabilization. Science.

[52] Rege N.K., Wickramasinghe N.P., Tustan A.N., Phillips N.F.B., Yee V.C., Ismail-Beigi F., Weiss M.A. (2018). Structure-based stabilization of insulin as a therapeutic protein assembly via enhanced aromatic-aromatic interactions. J. Biol. Chem..

[53] Gray H.B., Winkler J.R. (2015). Hole hopping through tyrosine/tryptophan chains protects proteins from oxidative damage. Proc. Natl. Acad. Sci. USA.

[54] Kunz P., Zinner K., Mücke N., Bartoschik T., Muyldermans S., Hoheisel J.D. (2018). The structural basis of nanobody unfolding reversibility and thermoresistance. Sci. Rep..

[55] Liu X., Taylor R.D., Griffin L., Coker S.F., Adams R., Ceska T., Shi J., Lawson A.D., Baker T. (2017). Computational design of an epitope-specific Keap1 binding antibody using hotspot residues grafting and CDR loop swapping. Sci. Rep..

[56] Kuroda D., Shirai H., Jacobson M.P., Nakamura H. (2012). Computer-aided antibody design. Protein Eng. Des. Sel..

[57] Duhoo Y., Roche J., Trinh T.T.N., Desmyter A., Gaubert A., Kellenberger C., Cambillau C., Roussel A., Leone P. (2017). Camelid nanobodies used as crystallization chaperones for different constructs of PorM, a component of the type IX secretion system from *Porphyromonas gingivalis*. Acta Crystallogr. F Struct. Biol. Commun..

[58] Kromann-Hansen T., Oldenburg E., Yung K.W., Ghassabeh G.H., Muyldermans S., Declerck P.J., Huang M., Andreasen P.A., Ngo J.C. (2016). A Camelid-derived Antibody Fragment Targeting the Active Site of a Serine Protease Balances between Inhibitor and Substrate Behavior. J. Biol. Chem..

[59] Van den Abbeele A., De Clercq S., De Ganck A., De Corte V., Van Loo B., Soror S.H., Srinivasan V., Steyaert J., Vandekerckhove J., Gettemans J. (2010). A llama-derived gelsolin single-domain antibody blocks gelsolin-G-actin interaction. Cell Mol. Life Sci..

[60] Pronk S., Pall S., Schulz R., Larsson P., Bjelkmar P., Apostolov R., Shirts M.R., Smith J.C., Kasson P.M., van der Spoel D. (2013). GROMACS 4.5: A high-throughput and highly parallel open source molecular simulation toolkit. Bioinformatics.

[61] Lindorff-Larsen K., Piana S., Palmo K., Maragakis P., Klepeis J.L., Dror R.O., Shaw D.E. (2010). Improved side-chain torsion potentials for the Amber ff99SB protein force field. Proteins.

[62] Bussi G., Donadio D., Parrinello M. (2007). Canonical sampling through velocity rescaling. J. Chem. Phys..

[63] Aragones J.L., Vega C. (2009). Plastic crystal phases of simple water models. J. Chem. Phys..

[64] Hess B. (2008). P-LINCS: A Parallel Linear Constraint Solver for Molecular Simulation. J. Chem. Theory Comput..

[65] Trott O., Olson A.J. (2010). AutoDock Vina: Improving the speed and accuracy of docking with a new scoring function, efficient optimization, and multithreading. J. Comput. Chem..

[66] Hatherley D., Lea S.M., Johnson S., Barclay A.N. (2014). Polymorphisms in the human inhibitory signal-regulatory protein alpha do not affect binding to its ligand CD47. J. Biol. Chem..

[67] Betz A.G., Neuberger M.S., Milstein C. (1993). Discriminating intrinsic and antigen-selected mutational hotspots in immunoglobulin V genes. Immunol. Today.

[68] Rogozin I.B., Kolchanov N.A. (1992). Somatic hypermutagenesis in immunoglobulin genes. II. Influence of neighbouring base sequences on mutagenesis. Biochim. Biophys. Acta.

[69] Guex N., Peitsch M.C. (1997). SWISS-MODEL and the Swiss-PdbViewer: An environment for comparative protein modeling. Electrophoresis.

[70] Breslin C., Hornyak P., Ridley A., Rulten S.L., Hanzlikova H., Oliver A.W., Caldecott K.W. (2015). The XRCC1 phosphate-binding pocket binds poly (ADP-ribose) and is required for XRCC1 function. Nucleic Acids Res..

