# Supplementary material for: Homology Modeling-Based in Silico Affinity Maturation Improves the Affinity of a Nanobody
Source: Int J Mol Sci. 2019 Aug 27;20(17):4187. doi: 10.3390/ijms20174187 (PMC6747709; doi:10.3390/ijms20174187)
Supplement: Supplementary file 1 [file ijms-20-04187-s001.pdf]

Homology Modeling-based in Silico Affinity Maturation Improves the Affinity of a Nanobody

Xin Cheng [1,2], Jiewen Wang [1,2], Guangbo Kang [1,2], Min Hu [1,2], Bo Yuan [1,2], Yingtian Zhang [1,2] and He Huang [1,2,*]

1 Department of Biochemical Engineering, School of Chemical Engineering & Technology, Tianjin University, Tianjin 300350, China
2 Key Laboratory of Systems Bioengineering (Ministry of Education), Tianjin University, Tianjin 300072, China
* Correspondence: huang@tju.edu.cn; Tel.: +0086-22-2740-3389

Supplementary Figures

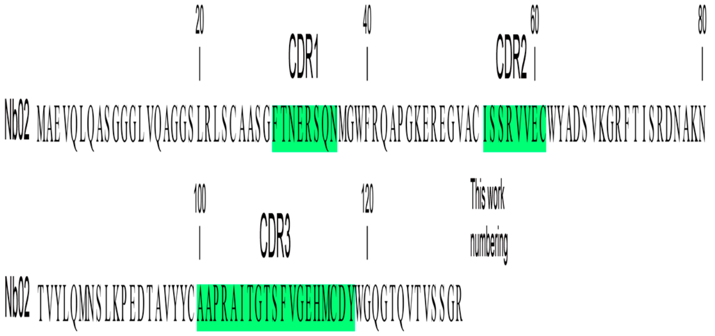

Figure S1. Evaluation results of molpdf and DOPE of 50 models. Five models: No.14, No.18, No.19, No.28, No.38 with lowest molpdf value were pre-selected. The model with lowest DOPE value within them, No.19, was selected as the optimum one.

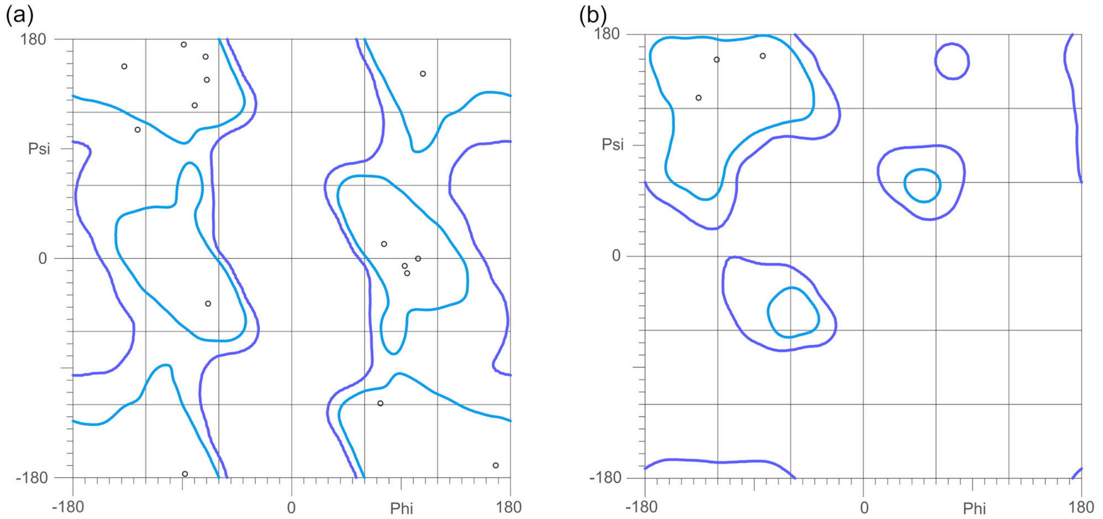

Figure S2. Ramachandran plot of the 3-D structure of Nb02. (**a**) Ramachandran plot of Gly; (**b**) Ramachandran plot of Pro.

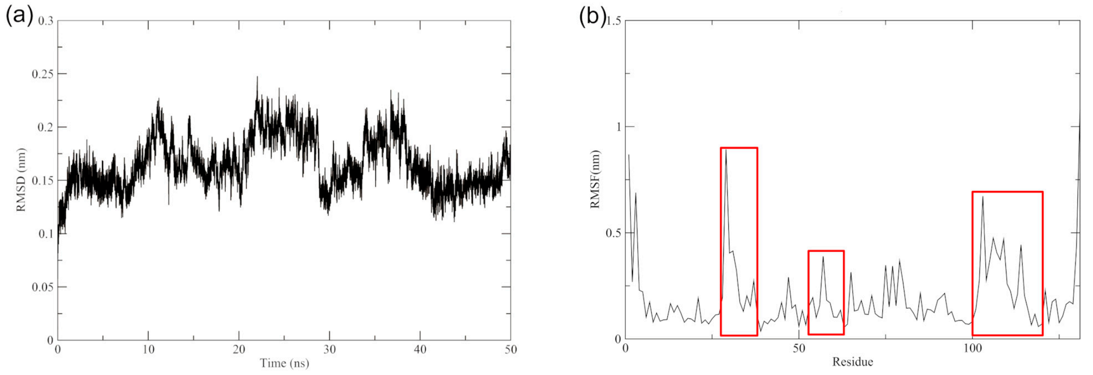

Figure S3. Analysis of Nb02 end simulation conformation. (**a**) Nb02 RMSD along the MD simulation; (**b**) Nb02 RMSF averaged over the same time interval.

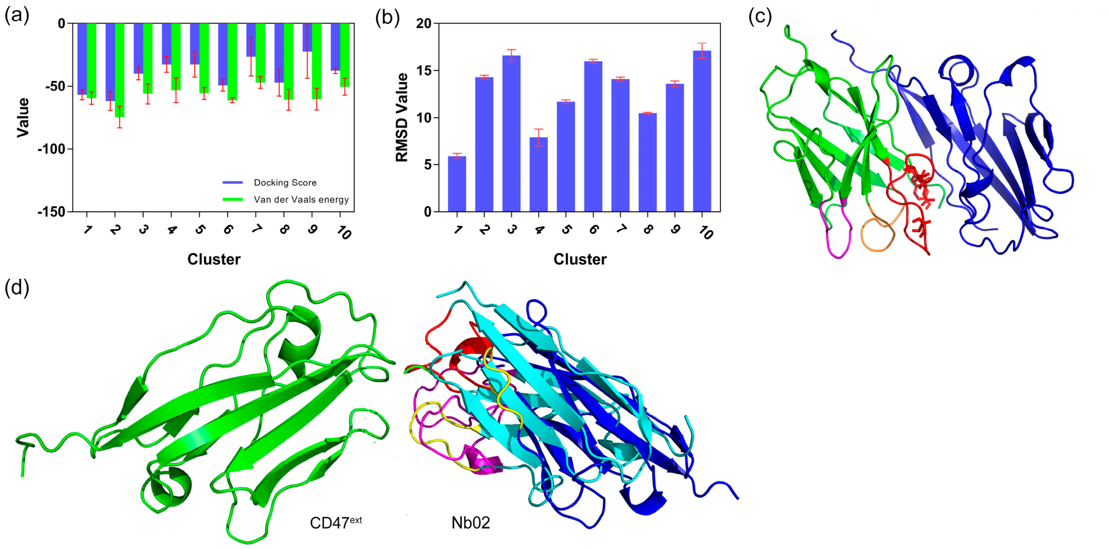

Figure S4. Analysis of end-docking structures. (**a**) values of docking score and *Van der Vaals* energy; (**b**) RMSD value of clusters; (**c**) pre-docking structure. The CD47^{ext} is in blue and Nb02 in green. CDRs are in yellow, purple and red, respectively; (**d**) Superimposed end-docking structure. Left structure represents CD47^{ext} and right for Nb02 (cyan represents the structure used in the text and blue represent another end-docking structure). CDRs are in red, yellow and magenta, respectively.

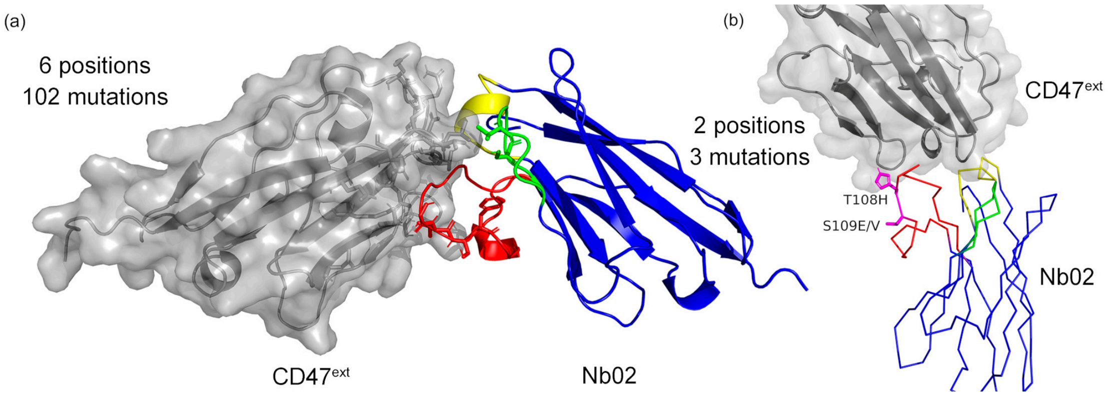

Figure S5. ADAPT round 1 screening leading to validated affinity-improved single-point mutants. (**a**) Proximity of virtually scanned residues to the CD47^{ext} epitope. (**b**) 3-D locations of single-point mutations with binding affinity improvement of over 10-fold relative to parental VHH. The antigen is shown as black/gray ribbon in a translucent molecular surface. The VHH is in blue, with CDR1 in yellow, CDR2 in green, and CDR3 in red, and their side chains shown as stick models. Models of

mutated side-chains are shown as magenta sticks.

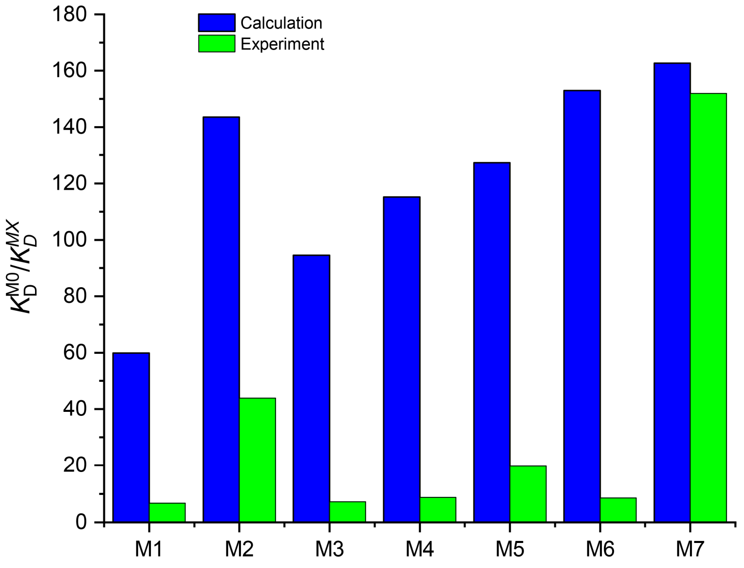

Figure S6. Comparison of the experimentally determined and calculated K_D^{M0}/K_D^{MX} ratios. The *K*D values were from Fig. 3 and SPR experiments.

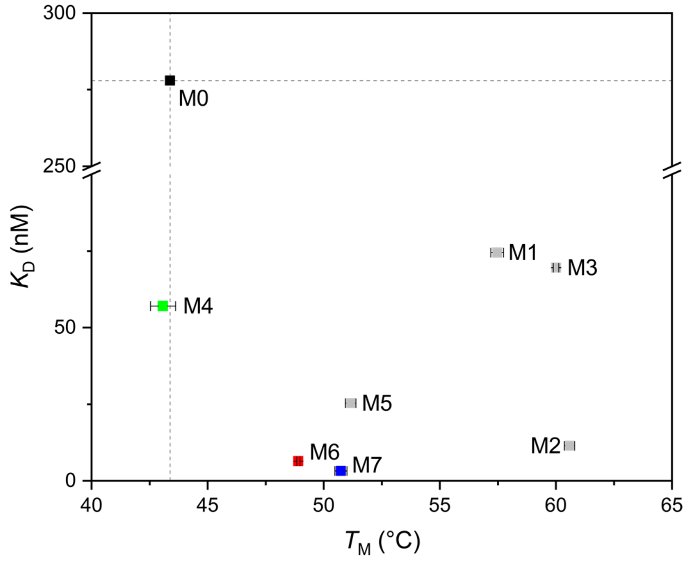

Figure S7. Comparison of the affinities and stabilities of the VHHs. The K_D values were from Figure 5 and the T_M values were from Fig. 6. The values of T_M are averages from five independent experiments and the error bars are SD.

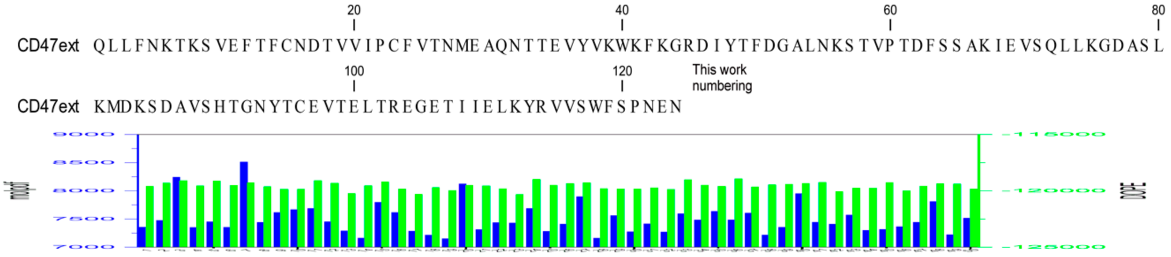

Figure S8. Amino acid sequences of the protein fragments used for in silico affinity maturation studies. The sequence of the VHH2 (GenBank accession number MK780744) used for experimental measurements in this work has a C-terminal His6 tag (HHHHHH) that was not included for in silico studies. The three CDR loops of the VHH are highlighted.

Supplementary Tables

Table S1: Consensus Z-scores for single mutants

	A	R	N	D	Q	E	G	H	I	L	K	M	F	S	T	W	Y	V
S55	−1.4	−2.7	−1.7	−2.2	−2.3	−1.4	−1.4	−1.0	−1.0	−2.1	−1.4	−1.6	−1.8		0.0	−1.6	−1.7	−1.4
V58	−1.0	−1.5	−1.7	−2.1	−1.6	−1.5	−1.6	−1.0	−1.4	−1.9	−1.7	−1.7	−1.0	−1.7	−1.7	−0.9	−1.0	
G107	−1.4	−2.1	−1.4	−1.5	−1.9	−1.8		−1.7	−2.2	−1.8	−1.8	−1.8	−1.7	−1.9	−2.1	−2.0	−1.9	−1.7
T108	−1.0	−1.4	0.0	−1.3	−1.0		−1.3	−2.3	−1.4	−1.0	−2.1	−1.4	−1.7	−1.0		−1.9	−1.8	−1.7
S109	1.4	−1.4	−1.6	−2.0	−1.4	−2.3	−2.0	−1.5	−1.4	−1.4	−1.6	0.0	−1.9		−1.4	−1.7	−1.4	−2.0
F110	−2.0	−2.2	−2.0	−1.3	−1.7	−1.6	−1.4	−1.0	−1.4	−1.7	−1.4	−1.4		−1.9	−1.4	−1.4	0.0	−1.6
